# Thyroid Hormone-Regulated Cardiac microRNAs are Predicted to Suppress Pathological Hypertrophic Signaling

**DOI:** 10.3389/fendo.2014.00171

**Published:** 2014-10-20

**Authors:** Rob Janssen, Marian J. Zuidwijk, Diederik W. D. Kuster, Alice Muller, Warner S. Simonides

**Affiliations:** ^1^Department of Physiology, VU University Medical Center, Institute for Cardiovascular Research, Amsterdam, Netherlands

**Keywords:** thyroid hormone, hyperthyroidism, microRNA, pathway analysis, physiological hypertrophy, pathological hypertrophy

## Abstract

Cardiomyocyte size in the healthy heart is in part determined by the level of circulating thyroid hormone (TH). Higher levels of TH induce ventricular hypertrophy, primarily in response to an increase in hemodynamic load. Normal cardiac function is maintained in this form of hypertrophy, whereas progressive contractile dysfunction is a hallmark of pathological hypertrophy. MicroRNAs (miRNAs) are important modulators of signal-transduction pathways driving adverse remodeling. Because little is known about the involvement of miRNAs in cardiac TH action and hypertrophy, we examined the miRNA expression profile of the hypertrophied left ventricle (LV) using a mouse model of TH-induced cardiac hypertrophy. C57Bl/6J mice were rendered hypothyroid by treatment with propylthiouracil and were subsequently treated for 3 days with TH (T3) or saline. T3 treatment increased LV weight by 38% (*p* < 0.05). RNA was isolated from the LV and expression of 641 mouse miRNAs was determined using Taqman Megaplex arrays. Data were analyzed using RQ-manager and DataAssist. A total of 52 T3-regulated miRNAs showing a >2-fold change (*p* < 0.05) were included in Ingenuity Pathway Analysis to predict target mRNAs involved in cardiac hypertrophy. The analysis was further restricted to proteins that have been validated as key factors in hypertrophic signal transduction in mouse models of ventricular remodeling. A total of 27 mRNAs were identified as *bona fide* targets. The predicted regulation of 19% of these targets indicates enhancement of physiological hypertrophy, while 56% indicates suppression of pathological remodeling. Our data suggest that cardiac TH action includes a novel level of regulation in which a unique set of TH-dependent miRNAs primarily suppresses pathological hypertrophic signaling. This may be relevant for our understanding of the progression of adverse remodeling, since cardiac TH levels are known to decrease substantially in various forms of pathological hypertrophy.

## Introduction

An increase in the hemodynamic load placed on the heart induces cardiomyocyte hypertrophy. The resulting increase in ventricular mass is aimed at normalizing wall stress and maintaining cardiac output. In the context of chronic pressure and/or volume overload, such as in hypertension, aortic stenosis, valvular disease, or following myocardial infarction, the initially adaptive response is associated with progressive contractile dysfunction and heart failure (1). This pathological remodeling is characterized by distinct changes in cardiac gene expression, increased cardiomyocyte apoptosis, and fibrosis. Typical phenotypic changes include a shift in expression from the fast contractile protein myosin heavy chain α (*Myh6*) to the slow β isoform (*Myh7*) (2), reduced expression of sarcoplasmic reticulum Ca^2+^-ATPase (*Serca2a*) (2, 3), and increased expression of fetal genes [e.g., α-skeletal actin, atrial natriuretic peptide (*Anp*), B-type natriuretic peptide (*Bnp*), deiodinase type III (*Dio3*) (4–6)].

In contrast, cardiac hypertrophy in response to increased levels of circulating thyroid hormone (TH) (hyperthyroidism) is not associated with adverse remodeling or impairment of contractility in both animals and human beings (7–9), in spite of the chronically increased hemodynamic load, which also in this case is the principal trigger of cardiomyocyte growth (10, 11). TH-induced remodeling is, therefore, considered physiological and is similar to physiological ventricular hypertrophy induced by pregnancy or chronic exercise training, and is equally reversible (12–14).

The distinction between stimuli and the related signal-transduction pathways that cause either pathological or physiological hypertrophy has not been without discussion. It has been presumed that the duration of the stimulus, i.e., chronic or intermittent, is the key difference determining the two forms of hypertrophy. Nevertheless, studies using intermittent pressure overload showed that although the resulting hypertrophy appeared to be adaptive, the cellular characteristics already showed all features of pathological remodeling (15). This suggests that certain aspects of the different stimuli and down-stream processes determine the phenotype of hypertrophy, rather than the duration of the exposure to the stimuli (16).

Gain- and loss-of-function studies have delineated numerous signal-transduction pathways which relay the various neurohumoral and mechanical signals that drive cardiac hypertrophy [see Ref. (16) for an extensive review]. There is considerable overlap and cross-talk between these pathways and although some pathways appear to mediate physiological, and others, pathological hypertrophy, the mechanisms underlying development of either one of these distinct phenotypes in response to chronic overload are not clear (16). In the case of TH, some effects of pathological signaling will be countered by direct transcriptional regulation of genes such as *Myh6, Myh7*, and *Serca2a* (7, 17), mediated by the TH receptors α1 or β1 (18). Both receptors are capable of mediating the transcriptional T3 effect, and they are also implicated in the non-genomic T3 effect involving stimulation of the phosphoinositide 3-kinase [PI3K (p110α)]-Akt pathway (19–21). However, additional mechanisms are expected to account for the full suppression of adverse remodeling.

Recent studies have shown an important role for microRNAs (miRNAs) in the regulation of cardiac gene expression by virtue of their ability to induce degradation of specific target mRNAs or reduce the efficiency of translation (22, 23). Although miRNAs are generally considered to fine-tune gene expression, critical roles of individual miRNAs have been established in various aspects of pathological cardiac hypertrophy (22–25). For example, miR-208a is required for overload-induced pathological remodeling, including cardiomyocyte hypertrophy, *Myh* isoform switching, and fibrosis (26, 27). Furthermore, forced expression of miR-208a induces pathological remodeling (27), whereas blocking of miR-208a attenuates hypertension-induced cardiac dysfunction (28).This miRNA is encoded in an intron of the *Myh6* gene, which is transcriptionally stimulated by TH. Consequently, and perhaps counter intuitively, expression of this “pathological” miRNA is increased in hyperthyroidism (27, 29). Little else is known about the TH-dependency of miRNA expression in the heart, despite the obvious potential of miRNA-mediated effects. However, studies in liver and skeletal muscle (30, 31) have identified a number of TH-responsive miRNAs, which are also known to play a role in remodeling adult cardiomyocytes and differentiating cardiomyocyte progenitor cells (32–35).

As a first step in elucidating a role of miRNAs in cardiac TH action, we set out to identify TH-responsive cardiac miRNAs using a mouse model of physiological hypertrophy induced by TH. To increase the sensitivity and to limit long-term secondary effects, TH-deficient mice were treated for 3 days with T3, the active form of TH. Target analysis of the 52 differentially regulated miRNAs indicated limited potentiation of pathways involved in physiological hypertrophy, but a marked suppression of pathways associated with pathological hypertrophy. Our data suggest that cardiac TH action includes a novel level of regulation in which a unique set of TH-dependent miRNAs suppresses pathological hypertrophic signaling.

## Materials and Methods

### Animals

Two groups of six male C57BL/6 mice (10–12 weeks, Charles Rivers) were used. Both groups were allowed *ad libitum* access to food containing PTU (Teklad + 0.15% propylthiouracil) for 44 days to induce hypothyroidism. Six mice were injected (IP) with a supra-physiological dose of 5 μg T3 in 20 μl saline at day 42, 43, and 44 (corresponding to 0.21–0.24 μg T3/g BW); the remaining six hypothyroid mice were injected with 20 μl saline. Animals were sacrificed at day 45. The heart was excised and left and right ventricle (LV, RV) and septum were dissected, weighed, immediately frozen in liquid nitrogen, and stored at −80°C. Tibia length was determined for normalization purposes. Plasma T3 levels were determined by AccuBind ELISA kits (Monobind Inc., Lake Forest, CA) according to the manufacturer’s instructions. Animals were housed individually and all experiments complied with the Guide for Care and Use of Laboratory Animals of the National Institutes of Health (NIH Publication no. 86-23, revised 1996) and were approved by the Institutional Animal Care and use Committees of VU University Medical Center Amsterdam.

### RNA isolation

Total RNA was isolated from approximately 20 mg LV using the mirVana Paris isolation kit (Ambion, Foster City, CA). To improve cell lysis, frozen samples were sliced into 10 μm sections using a cryostat. The tissue sections were dissolved in 2 ml cell disruption buffer. Three aliquots of 500 μl lysate were transferred to Eppendorf tubes and 500 μl of 2× denaturation solution, containing guanidinium thiocyanate, was added to each tube to prevent RNA degradation by cellular ribonucleases. Subsequently, an equal volume of acid-phenol:chloroform was added to separate RNA from DNA and protein (36). The aqueous phase was transferred to a fresh tube. To make sure there was no DNA or protein left, we repeated the previous step. Next, 1.25 volumes of 100% ethanol was added before each extract was transferred onto an individual filter cartridge, centrifuged, and washed. The bound RNA fraction was pretreated with DNAseI (Qiagen, Venlo, the Netherlands) before 105 μl elution buffer was added to each filter cartridge to collect the total RNA fraction. It should be noted that the capacity of the filter is limited, requiring the use of three cartridges per sample. RNA concentration and quality were measured either with the Nanodrop1000 (Thermo Scientific, Wilmington, DE) or the 2100 Bioanalyzer (Agilent Technologies, Santa Clara, CA). Samples were stored at −80°C, at which temperature miRNAs are stable (37).

### Quantitative real-time PCR

To analyze mRNA levels of the TH-regulated myosin heavy chains (*Myh6, Myh7*), 10 μl DNaseI-treated total RNA was transcribed to cDNA using Cloned AMV first strand cDNA synthesis (Invitrogen, Carlsbad, CA, USA). RNA concentration was adjusted to 12.5 ng/μl. Quantitative real-time polymerase chain reaction (qPCR) was performed using MESA GREEN qPCR MasterMix Plus for SYBR assay (Eurogentec, Seraing, Belgium) and specific primers (38) for *Myh6* (sense: 5′-GACCAGGCCAATGAGTACCG-3, antisense: 5′-GCCTAGCCAACTCCCCGTTC-3′) and *Myh7* (sense: 5′-CGCTCCACGCACCCTCACTT-3′, antisense: 5′-GTCCATCACC CCT GGAGAC-3′) in an Applied Biosystems model 7700 (Applied Biosystems, Foster City, CA). Expression levels of *Hprt* (sense: 5′-TCCCTGGTTAAGCAGTACAGCC-3′, antisense: 5′-CGAGAGGTCCTTTTCACCAGC-3′) were used for normalization. For comparison of the relative gene expression [2^(Ct HPRT – Ct target)^] of the two groups, a two-tailed Student *t*-test was performed to calculate *p* values.

### MicroRNA expression analysis

TaqMan miRNA Megaplex array (Applied Biosystems, Foster City, CA) was used to analyze miRNAs expression profiles. To synthesize cDNA, 3 μl RNA was added to 4.5 μl of RT-PCR reaction mix consisting of specially designed Megaplex RT primers (Rodent pool A or B, v3), dNTPs, MultiScribe reverse transcriptase, 10× reaction buffer, MgCl_2_, and RNase inhibitor. Subsequently, the mixture was subjected to 40 cycles for 2 min at 16°C, for 1 min at 42°C, and for 1 s at 50°C, followed by 5 min at 85°C and cooling to 4°C. A pre-amplification was performed to increase the cDNA quantity, thereby improving the sensitivity of miRNA detection (39). The pre-amplification mixture consisted of 2.5 μl RT product, 12.5 μl TaqMan PreAmp master mix, and Megaplex PreAmp primers. The mixture was incubated for 10 min at 95°C, 2 min at 55°C, 2 min at 72°C followed by 12 cycles of 15 s 95°C and 4 min at 60°C, inactivated for 10 min at 99.9°C, and cooled down to 4°C. Each product was diluted by adding 75 μl 0.1× TE (pH 8.0). After 9 μl diluted pre-amplified target cDNA was added to 450 μl 2× TaqMan Universal master mix (w/o amperase) and 441 μl nuclease free water, 100 μl mixture per lane was added to the TaqMan array card. The qPCR was performed on a 7900HT system with TLDA arrays. MammU6 and snoRNA202 were the most stable miRNAs and, therefore, chosen as endogenous controls. Comparative threshold (Ct) values were analyzed by SDS software v2.3 before being exported to RQ-manager v1.2 (Applied Biosystems). Data were next exported to DataAssist v3.0 (Applied Biosystems) for statistical analyses. MiRNAs with Ct’s above 38 were designated “not-expressed” and excluded from the analyses. Data were only considered when expression of a particular miRNA was observed in at least five mice of each group. A two-tailed Student *t*-test was used for two-group comparisons.

### Prediction of miRNA targets

To obtain an overview of the predicted miRNA targets and their role in biological processes, we analyzed significantly differentially regulated miRNAs with Ingenuity Systems Pathway Analysis software (IPA, Ingenuity Systems, www.ingenuity.com). Briefly, after analyzing the expression of all the detected miRNAs using DataAssist, a list of significantly differentially regulated miRNAs was uploaded to IPA. The uploaded miRNAs were analyzed using the microRNA Target Filter to find experimentally validated miRNA targets from Tarbase and miRecords, as well as highly predicted miRNA-mRNA pairing from Targetscan. Additionally, IPA includes peer-reviewed biological literature describing miRNA–target interactions. We focused only on miRNAs that were up- or downregulated by at least a fold change (FC) of >±2 and *p*-value of <0.05. Targets expressed in heart tissue were used for further *in silico* analysis.

## Results

### Left ventricular hypertrophy induced by T3 treatment

Hypothyroidism was induced using the established 40-day protocol of *ad libitum* access to food containing PTU (40). Indicative of the low plasma T3 levels and in agreement with earlier studies, growth was arrested in these animals (Table 1) (40). T3 treatment for three consecutive days resulted in a ~475-fold increase of plasma T3. Cardiac hypertrophy was evident from a 41% increase in total heart weight, and a 38% increase in weight of the LV free wall, which was used in all further analyses (Table 1).

**Table 1:** **Effects of short-term T3 treatment on BW, HW, and plasma T3 levels**.

	HO	HOT3
Anatomic data	
Body weight day 0 (g)	22.1 ± 0.5	22.1 ± 0.6
Body weight day 44 (g)	22.7 ± 0.4	22.6 ± 0.5
Total heart weight/TL (mg/cm)	41.3 ± 1.1	58.1 ± 0.6*
LV weight/TL (mg/cm)	24.0 ± 1.4	33.1 ± 1.2*
Thyroid hormone status	
T3 levels (nM)	0.1 ± 0.1	47.5 ± 4.2*

Twelve mice were allowed *ad libitum* access to food containing PTU for 44 days. Six randomly chosen mice were injected with T3 at day 42, 43, and 44. Controls received vehicle. Mice were sacrificed at day 45. Body weight remained unchanged during the experiment. Total heart weight increased by 41%, and LV weight increased by 38%. T3 treatment resulted in 475 times higher T3 levels in the hyperthyroid mice. Heart weight was corrected for TL, tibia length; HO, hypothyroid; HOT3, hyperthyroid. Values are means ± SEM; *p < 0.05.

**Figure 1:**
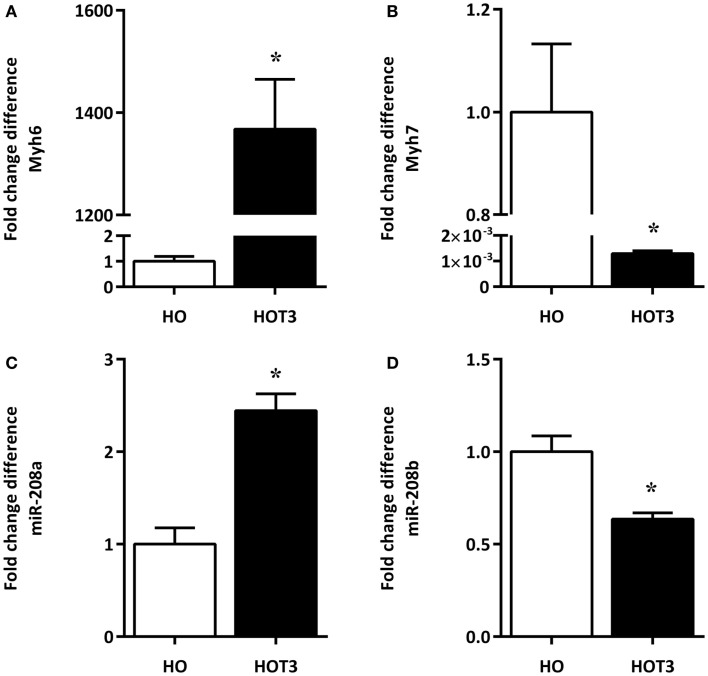
**The effects of T3 treatment on Myh-isoform expression and corresponding miRNAs**. RNA was isolated from the LV of six hypothyroid (HO) and six hyperthyroid (HOT3) mice. The low *Myh6* [**(A)**, open bar] and high *Myh7* mRNA levels [**(B)**, open bar] are indicative of the low cardiac T3 state in the hypothyroid animals. The TH-responsiveness of both genes was evident from the shift in expression of *Myh6* [**(A)**, solid bar] and *Myh7* [**(B)**, solid bar] as a result of T3 treatment. MiRNAs 208a and 208b are located within *Myh6* and *Myh7*. The expression pattern of miR-208a **(C)** and miR-208b **(D)** in these conditions is similar to the pattern of their host genes. HO set to 1, values are means ± SEM; *p < 0.05.

A total RNA fraction was isolated from approximately 20 mg LV tissue. Expression analysis of the TH-regulated *Myh6* and *Myh7* genes indicated the low cardiac T3 state in the hypothyroid animals, showing low *Myh6* and high *Myh7* mRNA levels (2). The TH-responsiveness of both genes was evident from the shift in expression of *Myh6* and *Myh7* as a result of T3 treatment (Figures 1A,B).

### Altered miRNA expression profile induced by T3 treatment

Using a platform based on stem-loop primers, we observed 45 significantly upregulated and 104 significantly downregulated miRNAs as a result of T3 treatment (Table S1 in Supplementary Material). Among these miRNAs, 208a and 208b are examples of miRNAs known to be up- and downregulated, respectively, in response to increasing TH levels (27, 29). Both miR-208a and 208b followed the expression of their corresponding host genes *Myh6* and *Myh7*, respectively (Figures 1C,D).

### Analysis of potential target mRNAs of T3-regulated miRNAs

**Figure 2:**
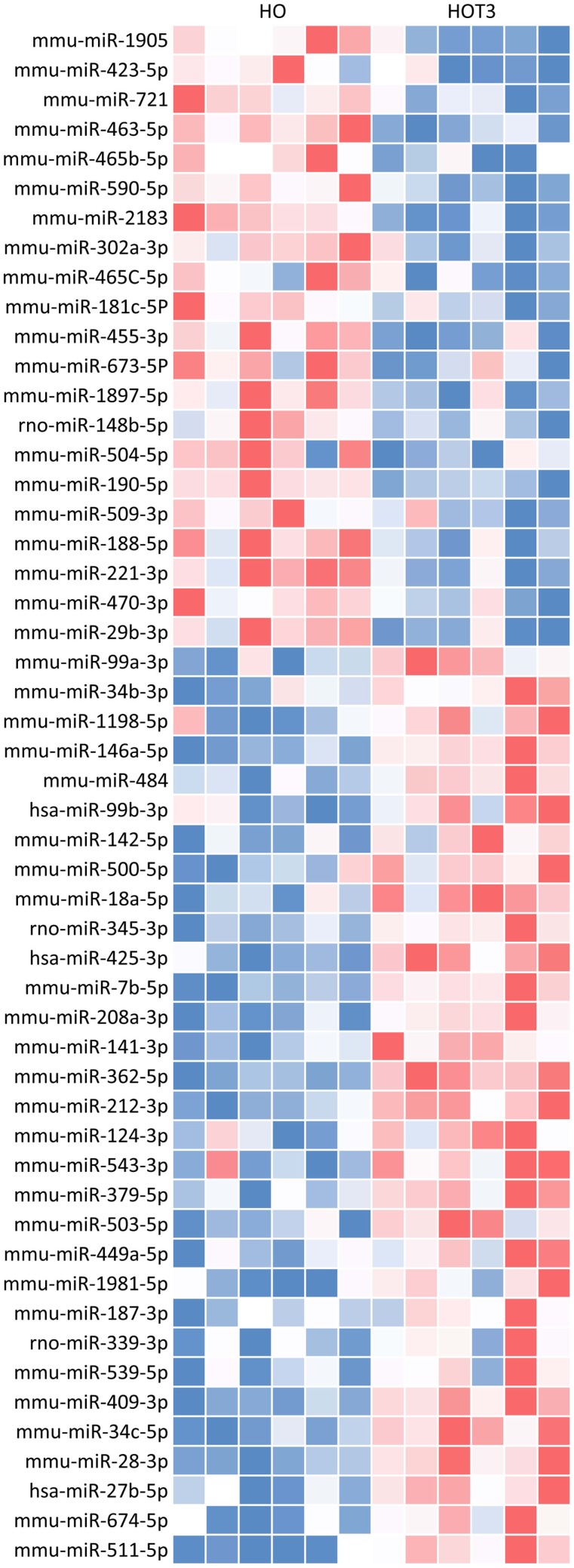
**T3 treatment for 3 days revealed a unique miRNA profile in the hypertrophied LV**. Expression data of 641 known mouse miRNAs were obtained with TaqMan Megaplex arrays (v3. A and B, rodent) and analyzed using RQ-manager (v1.2) and DataAssist (v3.0). The depicted heat map represents a unique profile of 52 differentially expressed miRNAs after 3 days of T3 treatment with a FC > ± 2 and a *p*-value < 0.05. Blue, low; red, high.

**Figure 3:**
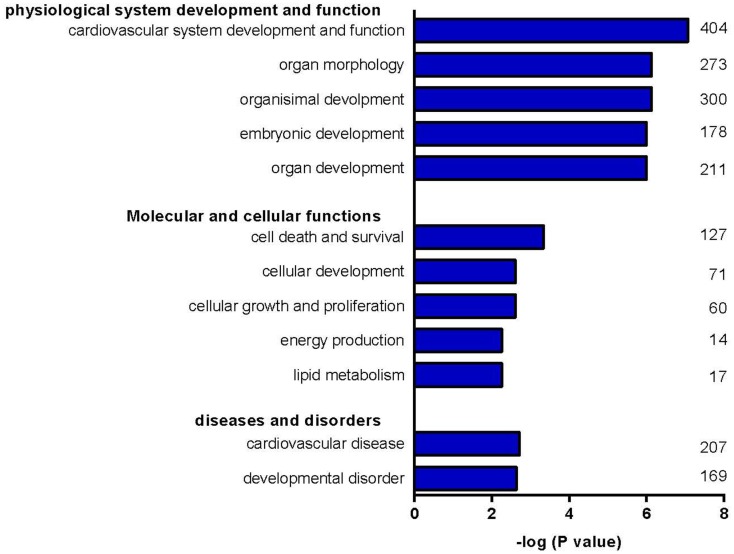
**Clustering of target mRNAs into biological groups**. Clustering of predicted target mRNAs into biological groups was performed with IPA. Predicted target mRNAs were represented in processes involved in “cardiovascular system development and function,” “cell death and survival,” “cellular growth and proliferation,” “energy metabolism,” and “lipid metabolism.” Numbers indicate the number of target mRNAs involved in the corresponding process.

Of the total of 149 differentially regulated miRNAs, 52 met the criteria of at least a twofold increase or a 50% decrease and having a *p*-value < 0.05. This group of 52 forms a unique signature of miRNAs differentially regulated after 3 days of T3 treatment (Figure 2). To test for potential target mRNAs, *in silico* target prediction was performed using a web-based entry tool, IPA. Thirty-one out of the 52 miRNAs had targeting information available in the Ingenuity database and could be used in the IPA analysis (Table S2 in Supplementary Material). The predicted interaction between miRNA and its target mRNA was based on sequence homology of the miRNA seed region and the target sequence present in the 3′ UTR of the mRNA. The analysis was furthermore restricted to mRNAs known to be expressed in heart tissue. In this way, a total of 3274 mRNAs were identified as potential targets of the group of 31 miRNAs. An IPA “core” analysis was subsequently performed to relate the predicted target mRNAs to known biological functions and processes. This revealed that the predicted target mRNAs were significantly represented in “cardiovascular system development and function,” “cell death and survival,” “cellular growth and proliferation,” “energy metabolism,” and “lipid metabolism” (Figure 3).

### Pathway analysis of potential target mRNAs involved in T3-induced cardiac hypertrophy

**Figure 4:**
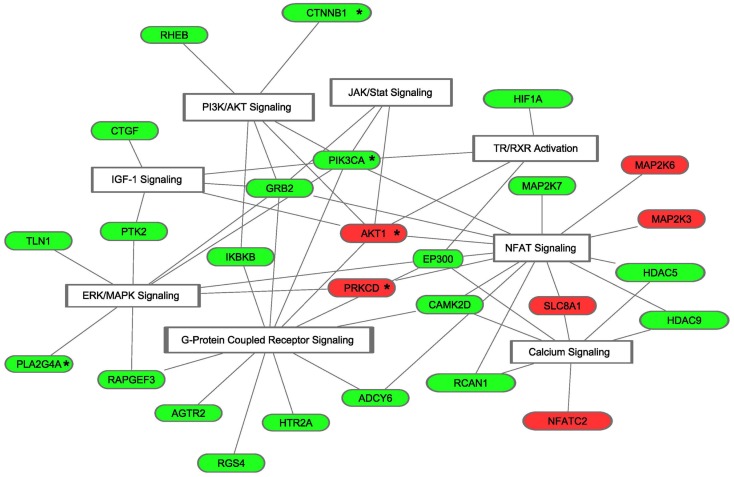
**Predicted target mRNAs involved in signature pathways of cardiac hypertrophy**. Of the 2312 mRNAs that were targeted by either up- or downregulated miRNAs, 27 *bona fide* targets were found to be involved in cardiac hypertrophy. The presented molecules are members of at least one of the eight selected signature transduction pathways involved in the development of cardiac hypertrophy. Red, the corresponding miRNA was shown to be downregulated in HOT3, which resulted in a predicted upregulation of its target mRNA; green, the corresponding miRNA was shown to be upregulated in hyperthyroid LV, which resulted in a predicted downregulation of its target mRNA. “*” Indicates target mRNAs involved in physiological hypertrophy.

We next focused our analyses on target mRNAs encoding proteins which have been shown to be critical components of hypertrophic signaling pathways, i.e., the IGF-1-, NFAT-, Ca^2+^-, GPCR-, JAK/STAT-, PI3K/Akt-, ERK/MAPK-, and TH-signaling pathways (16) and which have been validated in mouse models of pathological and/or physiological hypertrophy. Analysis was furthermore limited to mRNAs that are solely targeted by either up- or downregulated miRNAs, so that the effect of miRNA regulation on the expression of these signaling components is unequivocal. This approach resulted in 27 candidates (Figure 4). In depth analyses using IPA and additional literature searches revealed that the predicted regulation of 5 of these targets (19%) results in enhancement of physiological hypertrophy, whereas regulation of 15 of these targets (56%) results in suppression of pathological remodeling (Table 2).

**Table 2:** **Predicted interaction of T3-induced miRNA expression and hypertrophic signaling pathways**.

mRNA	miR		Induction	Reference
**UPREGULATED TARGET MRNAS INVOLVED IN PATHOLOGICAL HYPERTROPHY**				
*Map2k3*	590	Dominant negative *Map2k3* increases hypertrophy	TAC, AngII, Iso	(41)
*Map2k6*	29b	Dominant negative *Map2k6* increases hypertrophy	TAC, AngII, Iso	(41)
*Slc8a1*	721	uced levels of *Slc8a1* caused hypertrophy	TAC	(42)
**DOWNREGULATED TARGET MRNAS INVOLVED IN PATHOLOGICAL HYPERTROPHY**				
*Agtr2*	539	Chronic loss of *Agtr2* attenuates left ventricular hypertrophy	AngII	(43)
*Camk2d*	539, 7b	Inhibition prevents maladaptive remodeling	TAC, Iso	(44, 45)
*Ctgf*	124, 212, 18a	Inhibition attenuates LV remodeling in pressure overload-induced heart failure	TAC, AngII	(46, 47)
*Ep300*	212	Specific reduction of *Ep300* content or activity diminishes stress-induced hypertrophy	TAC	(48, 49)
*Grb2*	124, 141	*Grb2*^+/−^ mice showed a reduced hypertrophic response in response to overload	TAC	(50)
*Hif-1*α	18a	Deletion of *Hif-1*α prevents hypertrophy	TAC	(51)
*Htr2a*	34c	Blockade of *Htr2a* has a beneficial effect on the development of hypertrophy	TAC	(52)
*Ikbkb*	503	*Ikbkb* negatively regulates the anti-hypertrophic action of Irf7/Nf-kappa B in pathological hypertrophy	TAC, Pe, AngII	(53, 54)
*Ptk2/Fak*	379	uced levels of *Ptk2* were accompanied by prevention, as well as reversal, of load- induced LV hypertrophy	TAC	(55)
*Rapgef3*	539, 721	KO reduces β-adrenergic stimulation-induced hypertrophy	TAC	(56)
*Rheb*	141	*Rheb* activates mTORC1 signaling-dependent hypertrophy	TAC, MI	(57)
*Tln1*	124, 503	uction of *Tln1* expression improves cardiac remodeling	TAC	(58)
**UPREGULATED TARGET MRNA INVOLVED IN PHYSIOLOGICAL HYPERTROPHY**				
*Akt*	302	Increased *Akt* is involved in physiological hypertrophy	MI, Ex	(16, 59, 60)
*Prkcd*	181c	*Prkcd* activates non-pathological hypertrophy	MI	(61)
**DOWNREGULATED TARGET MRNA INVOLVED IN PHYSIOLOGICAL HYPERTROPHY**				
*Ctnnb1*	141	Down regulation is required for adaptive remodeling	AngII	(62)
*Pik3ca*	124	Constitutively active *Pik3ca*/*p110*α increases the hypertrophic response in TAC;, is activated in physiological hypertrophy	TAC, Ex	(63, 64)
*Pla2g4a*	543	Hypertrophic growth is increased in *Pla2g4a*^−/−^ in TAC; increasing *Igf-1*	TAC	(65)

*IPA and additional literature were used to evaluate the role of 27 *bona fide* target mRNAs in the development of cardiac hypertrophy (see Figure 4). Twenty of the initial 27 target mRNAs were shown to be validated in mouse models for cardiac hypertrophy, as indicated in the table, together with the relevant literature and description of the principal findings. These models included transverse (ascending) aortic constriction (TAC), LV remodeling following myocardial infarction (MI), and Angiotensin II (AngII), isoproterenol (Iso), and phenylephrine (Pe) induced pathological hypertrophy, and exercise (Ex) induced physiological hypertrophy. The identified T3-regulated miRNAs are depicted in green (reduced expression) or red (increased expression). The direction of change of the predicted target mRNAs is similarly color coded. Fifteen target mRNAs are predicted to be regulated by the indicated miRNAs in such a way that pathological signaling is suppressed. For example, *Map2k3* has been shown in a TAC model to suppress pathological remodeling. Consequently, its upregulation by decreased miR-590 expression is considered to limit pathological signaling. Likewise, *Camk2d* is required for TAC-induced pathological remodeling and its suppression by the upregulated miRNAs 539 and 7b is again considered to limit pathological signaling. The remaining five target mRNAs are regulated in such a way that physiological signaling is enhanced*.

## Discussion

In this paper, we describe an unbiased *in silico* approach to analyze differentially expressed cardiac miRNAs in the physiologically hypertrophied mouse heart as a result of T3 treatment. Our data show that next to the known extra-cardiac effects of T3 on the heart due to an increase in hemodynamic load (10, 11), and modulation of TH-regulated genes (2, 3), a T3-dependent miRNA profile is expressed which is predicted to primarily suppress pathways involved in pathological hypertrophy, with some enhancement of pathways involved in physiological hypertrophy.

We chose to use hypothyroid mice treated for only 3 days with T3 to optimize the identification of T3-responsive miRNAs. The reciprocal regulation of the TH-regulated myosin genes, *Myh6* and *Myh7*, and the 41% increase in heart weight are in line with previous findings (14, 66, 67). Earlier studies established that the T3-induced increase in heart weight is caused by an increase in cell size and is not accompanied by fibrotic deposition (68, 69). Some of the observed changes in the miRNA expression profile of the hypertrophied LV, following T3 treatment, also confirmed earlier results. The observed reciprocal expression of the miRNAs 208a and 208b, which originate from introns of *Myh6* and *Myh7*, respectively, has been reported before (27, 29). As already mentioned in the Section “Introduction,” expression of miR-208a has been implicated in the development of pathological hypertrophy. Mir-208a targets the Thyroid Hormone Receptor Associated Protein 1 (*Thrap1*) (26, 27, 70), which is a component of the TH-receptor/co-factor complex that mediates transcriptional regulation of T3-dependent genes (26, 27, 71, 72). Increased miR-208a expression is, therefore, expected to decrease T3 action, because *Thrap1* expression will be decreased. However, our data suggest that in the presence of high levels of T3, the effect of miR-208a on the efficiency of T3-regulated gene expression and pathological signaling is minimal, since it does not prevent T3-induced gene expression nor does it result in a pathological phenotype under this condition. Also, in line with earlier results, the cardiac miRNAs, miR-206 and miR-1, which were shown to be downregulated by TH in liver and skeletal muscle (30, 31), were found to be suppressed by T3 treatment in the present study.

Furthermore, our results are consistent with data obtained using a model of exercise-induced cardiac hypertrophy (73), showing a significant downregulation of miR-26b, 27a, and 143 after 7 days of exercise. Moreover, a trend toward downregulation of miR-195 and 499 was observed in that study, where we found significant downregulation of both miRNAs after 3 days of T3 treatment. Exercise-induced physiological cardiac growth has been considered similar to TH-induced physiological hypertrophy (16), and our data suggest that this similarity may involve regulation by miRNAs.

In-depth analysis of our set of 149 differentially expressed miRNAs revealed 52 with a FC > ±2 and a *p*-value < 0.05, which formed a miRNA signature unique for short-term T3 treatment in the LV. To test for potential target mRNAs, *in silico* target prediction was performed using IPA. Thirty-one of the 52 had targeting information available in the database, resulting in 3274 confirmed or highly predicted targets. Core analysis included in IPA was used to interpret the target mRNA data in the context of biological functions and processes. Besides “cardiovascular system development and function,” processes in which target mRNAs are known to be involved included “cell death and survival,” “cellular growth and proliferation,” “energy metabolism,” and “lipid metabolism.” These findings are in accordance with the concept that TH plays a determining role in cell proliferation, differentiation, and metabolism (74, 75).

Next, the analysis was limited to target mRNAs that were solely targeted by either up- or downregulated miRNAs, so that the effect of miRNA regulation on the expression of these signaling components was unequivocal, resulting in 2312 target mRNAs. Subsequently, we searched for target mRNAs involved in cardiac hypertrophy pathways. Signature cardiac hypertrophy pathways, such as IGF-1-, PI3K/Akt-, JAK/STAT-, and TH-signaling pathways, are mostly associated with physiological hypertrophy, whereas GPCR-, Ca^2+^-, ERK/MAPK-, and NFAT signaling pathways are associated with pathological hypertrophy (16). Analysis revealed 27 target mRNAs that were indicated by IPA to be part of at least one of the signature pathways mentioned above and have been validated in models of pathological and/or physiological hypertrophy. In the setting of T3-induced hypertrophy, the predicted regulation of 19% of these targets indicates enhancement of physiological hypertrophy, while 56% indicates suppression of pathological remodeling.

For instance, *Hif1*α and *Camk2d* are upregulated in both human and mouse pathological hypertrophy (51, 76). Inactivation of *Hif1*α in TAC mouse models resulted in a beneficial effect preventing cardiac growth (51, 77). Transgenic mouse models overexpressing *Camk2d*, the predominant isoform expressed in the heart, established the involvement of *Camk2d* in pathological hypertrophy (78). Specific inhibition of C*amk2d* attenuated cardiac growth in response to several hypertrophic stimuli (44, 45, 79). It was consequently suggested that target-specific inhibition of *Camk2d* may be a useful treatment of cardiac hypertrophy (80). Another example is connective tissue growth factor, *Ctgf*, which in the present study is targeted by three upregulated miRNAs, miR-212, 124, and 18a, suggesting a strong downregulation. It has been reported that while the initial overexpression of *Ctgf* activates *Akt*, triggering adaptive hypertrophy, the prolonged *Ctgf* overexpression eventually leads to heart failure (46, 81). These, and other target mRNAs listed in Table 2 are predicted by IPA to be downregulated by T3-dependent miRNAs, suggesting that fine-tuning by these miRNAs attenuates the development of pathological hypertrophy.

Likewise, it has been described that increased expression of the serine/threonine kinase *Akt* results in cell growth (82), both in pathological and in physiological hypertrophy (83). Recent studies using *Akt* knockout mouse models suggest that *Akt* is more likely to be required for physiological hypertrophy than for pathological hypertrophy (59). It has been shown that short-term activation of *Akt* is associated with cardiac protection (84), utilization of glucose, instead of free fatty acids, and improvement of cardiac contractility (85). For *Ctnnb1*/Beta-catenin, a key molecule in pathways associated with cardiac hypertrophy, it has been shown that decreased expression levels are important for the process of adaptive left ventricular remodeling (62), suggesting that the predicted fine-tuning of these target mRNAs enhances physiological hypertrophy.

Although we limited our analyses to miRNAs that were strongly responsive to T3 treatment, and to target mRNAs that have been validated in mouse models, future studies should be aimed at confirming the predicted regulation of the target mRNAs and their proteins. At present, our data suggest that cardiac T3-action includes a novel level of regulation in which a unique set of T3-dependent miRNAs enhances some aspects of physiological hypertrophy, but primarily suppresses pathological hypertrophic signaling. This suggests a mechanism that may in part explain why cardiac hypertrophy due to high circulating T3 levels does not progress to dysfunction, in spite of the chronic hemodynamic overload, which in the context of cardiovascular disease is the principal trigger of pathological hypertrophy (10). This proposed mechanism may be relevant for our understanding of the progression of adverse remodeling. As in other severe illnesses, heart failure is often associated with reduced plasma T3 levels, the so-called non-thyroidal illness syndrome (86). Clinical studies have shown that the extent of the reduction is an independent predictor of mortality in patients with chronic heart failure (87, 88). In addition, we and others have shown that increased cardiac activity of the TH inactivating enzyme, *Dio3*, results in a local hypothyroid condition in the pathologically remodeled heart due to pressure overload or following myocardial infarction (38, 89, 90). Indeed, alterations in gene expression in the failing heart are to some extent similar to the genetic profile of a hypothyroid heart (4). Taken together, this suggests that reduced T3-signaling in the hemodynamically overloaded heart will aggravate adverse remodeling by releasing the T3-dependent miRNA brake on these pathological signaling pathways. Manipulation of these miRNAs may be an additional option in the ongoing development of miRNA-based therapeutics for the treatment of heart failure (91, 92).

## Conflict of Interest Statement

The authors declare that the research was conducted in the absence of any commercial or financial relationships that could be construed as a potential conflict of interest.

## Supplementary Material

The Supplementary Material for this article can be found online at http://www.frontiersin.org/Journal/10.3389/fendo.2014.00171/abstract

## References

[1] Sutton MGSJ Sharpe N. Left ventricular remodeling after myocardial infarction: pathophysiology and therapy. Circulation (2000) 101:2981–8. 10.1161/01.CIR.101.25.2981 10869273

[2] Ojamaa K Kenessey A Shenoy R Klein I Gerdes AM Iervasi G Thyroid hormone metabolism and cardiac gene expression after acute myocardial infarction in the rat. Am J Physiol Endocrinol Metab (2000) 279(6):E1319–24. 11093920 10.1152/ajpendo.2000.279.6.E1319

[3] Bers DM Eisner DA Valdivia HH. Sarcoplasmic reticulum Ca2+ and heart failure: roles of diastolic leak and Ca2+ transport. Circ Res (2003) 93:487–90. 10.1161/01.RES.0000091871.54907.6B 14500331

[4] Pol CJ Muller A Simonides WS. Cardiomyocyte-specific inactivation of thyroid hormone in pathologic ventricular hypertrophy: an adaptative response or part of the problem? Heart Fail Rev (2010) 15:133–42. 10.1007/s10741-008-9133-7 19107595 PMC2820687

[5] Dirkx E da Costa Martins P De Windt LJ. Regulation of fetal gene expression in heart failure. Biochim Biophys Acta (2013) 1832(12):2414–24. 10.1016/j.bbadis.2013.07.023 24036209

[6] Razeghi P Young ME Alcorn JL Moravec CS Frazier OHH Taegtmeyer H. Metabolic gene expression in fetal and failing human heart. Circulation (2001) 104:2923–31. 10.1161/hc4901.100526 11739307

[7] Klein I Ojamaa K. Thyroid hormone and the cardiovascular system. N Engl J Med (2001) 344:501–9. 10.1056/NEJM200102153440707 11172193

[8] Ching GW Franklyn J Stallard TJ Daykin J Sheppard MC Gammage MD. Cardiac hypertrophy as a result of long-term thyroxine therapy and thyrotoxicosis. Heart (1996) 75:363–8. 10.1136/hrt.75.4.363 8705762 PMC484311

[9] Rodondi N Newman AB Vittinghoff E de Rekeneire N Satterfield S Harris TB Subclinical hypothyroidism and the risk of heart failure, other cardiovascular events, and death. Arch Intern Med (2005) 165:2460–6. 10.1001/archinte.165.21.2460 16314541

[10] Danzi S Klein I. Thyroid hormone and blood pressure regulation. Curr Hypertens Rep (2003) 5:513–20. 10.1007/s11906-003-0060-7 14594573

[11] Trivieri MG Oudit GY Sah R Kerfant B-G Sun H Gramolini AO Cardiac-specific elevations in thyroid hormone enhance contractility and prevent pressure overload-induced cardiac dysfunction. Proc Natl Acad Sci U S A (2006) 103:6043–8. 10.1073/pnas.0601072103 16595628 PMC1426242

[12] Biondi B Palmieri EA Fazio S Cosco C Nocera M Saccà L Endogenous subclinical hyperthyroidism affects quality of life and cardiac morphology and function in young and middle-aged patients. J Clin Endocrinol Metab (2000) 85:4701–5. 10.1210/jcem.85.12.7085 11134131

[13] Klein I Hong C. Effects of thyroid hormone on cardiac size and myosin content of the heterotopically transplanted rat heart. J Clin Invest (1986) 77:1694–8. 10.1172/JCI112488 2939104 PMC424576

[14] Kuzman JA O’Connell TD Gerdes AM. Rapamycin prevents thyroid hormone-induced cardiac hypertrophy. Endocrinology (2007) 148:3477–84. 10.1210/en.2007-0099 17395699

[15] Perrino C Naga Prasad SV Mao L Noma T Yan Z Kim H-S Intermittent pressure overload triggers hypertrophy-independent cardiac dysfunction and vascular rarefaction. J Clin Invest (2006) 116:1547–60. 10.1172/JCI25397 16741575 PMC1464895

[16] Bernardo BC Weeks KL Pretorius L McMullen JR. Molecular distinction between physiological and pathological cardiac hypertrophy: experimental findings and therapeutic strategies. Pharmacol Ther (2010) 128:191–227. 10.1016/j.pharmthera.2010.04.005 20438756

[17] Dillmann W. Cardiac hypertrophy and thyroid hormone signaling. Heart Fail Rev (2010) 15:125–32. 10.1007/s10741-008-9125-7 19125327 PMC2820695

[18] Bassett JHD Harvey CB Williams GR. Mechanisms of thyroid hormone receptor-specific nuclear and extra nuclear actions. Mol Cell Endocrinol (2003) 213:1–11. 10.1016/j.mce.2003.10.033 15062569

[19] Kenessey A Ojamaa K. Thyroid hormone stimulates protein synthesis in the cardiomyocyte by activating the Akt-mTOR and p70S6K pathways. J Biol Chem (2006) 281:20666–72. 10.1074/jbc.M512671200 16717100

[20] Cao X Kambe F Moeller LC Refetoff S Seo H. Thyroid hormone induces rapid activation of Akt/protein kinase B-mammalian target of rapamycin-p70S6K cascade through phosphatidylinositol 3-kinase in human fibroblasts. Mol Endocrinol (2005) 19:102–12. 10.1210/me.2004-0093 15388791

[21] Martin NP Fernandez de Velasco EM Mizuno F Scappini EL Gloss B Erxleben C A rapid cytoplasmic mechanism for PI3 kinase regulation by the nuclear thyroid hormone receptor, TRβ, and genetic evidence for its role in the maturation of mouse hippocampal synapses in vivo. Endocrinology (2014) 155:3713–24. 10.1210/en.2013-2058 24932806 PMC4138568

[22] Thum T Catalucci D Bauersachs J. MicroRNAs: novel regulators in cardiac development and disease. Cardiovasc Res (2008) 79:562–70. 10.1093/cvr/cvn137 18511432

[23] Van Rooij E Sutherland LB Liu N Williams AH McAnally J Gerard RD A signature pattern of stress-responsive microRNAs that can evoke cardiac hypertrophy and heart failure. Proc Natl Acad Sci U S A (2006) 103:18255–60. 10.1073/pnas.0608791103 17108080 PMC1838739

[24] Da Costa Martins P Salic K Gladka MM Armand A-S Leptidis S El Azzouzi H MicroRNA-199b targets the nuclear kinase Dyrk1a in an auto-amplification loop promoting calcineurin/NFAT signalling. Nat Cell Biol (2010) 12:1220–7. 10.1038/ncb2126 21102440

[25] Divakaran V Mann DL. The emerging role of microRNAs in cardiac remodeling and heart failure. Circ Res (2008) 103:1072–83. 10.1161/CIRCRESAHA.108.183087 18988904 PMC3982911

[26] van Rooij E Sutherland LB Qi X Richardson J Hill J Olson EN. Control of stress-dependent cardiac growth and gene expression by a microRNA. Science (2007) 316:575–9. 10.1126/science.1139089 17379774

[27] Callis TE Pandya K Seok HY Tang R-H Tatsuguchi M Huang Z-P MicroRNA-208a is a regulator of cardiac hypertrophy and conduction in mice. J Clin Invest (2009) 119:2772–86. 10.1172/JCI36154 19726871 PMC2735902

[28] Montgomery RL Hullinger TG Semus HM Dickinson B Seto G Lynch JM Therapeutic inhibition of miR-208a improves cardiac function and survival during heart failure. Circulation (2011) 124(14):1537–47. 10.1161/CIRCULATIONAHA.111.030932 21900086 PMC3353551

[29] Diniz GP Takano AP Barreto-Chaves MLM. MiRNA-208a and miRNA-208b are triggered in thyroid hormone-induced cardiac hypertrophy - role of type 1 angiotensin II receptor (AT1R) on miRNA-208a/α-MHC modulation. Mol Cell Endocrinol (2013) 374:117–24. 10.1016/j.mce.2013.04.010 23623871

[30] Dong H Paquette M Williams A Zoeller RT Wade M Yauk C. Thyroid hormone may regulate mRNA abundance in liver by acting on microRNAs. PLoS One (2010) 5:e12136. 10.1371/journal.pone.0012136 20808432 PMC2921333

[31] Visser WE Heemstra K Swagemakers SM Ozgür Z Corssmit EP Burggraaf J Physiological thyroid hormone levels regulate numerous skeletal muscle transcripts. J Clin Endocrinol Metab (2009) 94:3487–96. 10.1210/jc.2009-0782 19567520

[32] Bagnall RD Tsoutsman T Shephard RE Ritchie W Semsarian C. Global MicroRNA profiling of the mouse ventricles during development of severe hypertrophic cardiomyopathy and heart failure. PLoS One (2012) 7:e44744. 10.1371/journal.pone.0044744 23024758 PMC3443088

[33] Shan Z-X Lin Q-X Fu Y-H Deng C-Y Zhou Z-L Zhu J-N Upregulated expression of miR-1/miR-206 in a rat model of myocardial infarction. Biochem Biophys Res Commun (2009) 381:597–601. 10.1016/j.bbrc.2009.02.097 19245789

[34] Sluijter JPG van Mil A van Vliet P Metz CHG Liu J Doevendans P MicroRNA-1 and -499 regulate differentiation and proliferation in human-derived cardiomyocyte progenitor cells. Arterioscler Thromb Vasc Biol (2010) 30:859–68. 10.1161/ATVBAHA.109.197434 20081117

[35] Vo NK Dalton RP Liu N Olson EN Goodman RH. Affinity purification of microRNA-133a with the cardiac transcription factor, Hand2. Proc Natl Acad Sci U S A (2010) 107:19231–6. 10.1073/pnas.1013162107 20974915 PMC2984217

[36] Chomczynski P Sacchi N. Single-step method of RNA isolation by acid guanidinium thiocyanate-phenol-chloroform extraction. Anal Biochem (1987) 159:156–9. 10.1006/abio.1987.9999 2440339

[37] Mraz M Malinova K Mayer J Pospisilova S. MicroRNA isolation and stability in stored RNA samples. Biochem Biophys Res Commun (2009) 390:1–4. 10.1016/j.bbrc.2009.09.061 19769940

[38] Pol CJ Muller A Zuidwijk MJ van Deel ED Kaptein E Saba A Left-ventricular remodeling after myocardial infarction is associated with a cardiomyocyte-specific hypothyroid condition. Endocrinology (2011) 152:669–79. 10.1210/en.2010-0431 21159857

[39] Chen Y Gelfond JL McManus LM Shireman PK. Reproducibility of quantitative RT-PCR array in miRNA expression profiling and comparison with microarray analysis. BMC Genomics (2009) 10:407. 10.1186/1471-2164-10-407 19715577 PMC2753550

[40] Bianco AC Anderson G Forrest D Galton VA Gereben B Kim BW American thyroid association guide to investigating thyroid hormone economy and action in rodent and cell models. Thyroid (2014) 24:88–168. 10.1089/thy.2013.0109 24001133 PMC3887458

[41] Braz JC Bueno OF Liang Q Wilkins BJ Dai Y-S Parsons S Targeted inhibition of p38 MAPK promotes hypertrophic cardiomyopathy through upregulation of calcineurin-NFAT signaling. J Clin Invest (2003) 111:1475–86. 10.1172/JCI17295 12750397 PMC155046

[42] Takimoto E Yao A Toko H Takano H Shimoyama M Sonoda M Sodium calcium exchanger plays a key role in alteration of cardiac function in response to pressure overload. FASEB J (2002) 16:373–8. 10.1096/fj.01-0735com 11874986

[43] Ichihara S Senbonmatsu T Price E Ichiki T Gaffney FA Inagami T. Angiotensin II type 2 receptor is essential for left ventricular hypertrophy and cardiac fibrosis in chronic angiotensin II-induced hypertension. Circulation (2001) 104:346–51. 10.1161/01.CIR.104.3.346 11457756

[44] Chang L Zhang J Tseng Y-H Xie C-Q Ilany J Brüning JC Rad GTPase deficiency leads to cardiac hypertrophy. Circulation (2007) 116:2976–83. 10.1161/CIRCULATIONAHA.107.707257 18056528 PMC4207362

[45] Li C Cai X Sun H Bai T Zheng X Zhou XW The δA isoform of calmodulin kinase II mediates pathological cardiac hypertrophy by interfering with the HDAC4-MEF2 signaling pathway. Biochem Biophys Res Commun (2011) 409:125–30. 10.1016/j.bbrc.2011.04.128 21554860 PMC3113443

[46] Panek AN Posch MG Alenina N Ghadge SK Erdmann B Popova E Connective tissue growth factor overexpression in cardiomyocytes promotes cardiac hypertrophy and protection against pressure overload. PLoS One (2009) 4:e6743. 10.1371/journal.pone.0006743 19707545 PMC2727794

[47] Szabó Z Magga J Alakoski T Ulvila J Piuhola J Vainio L Connective tissue growth factor inhibition attenuates left ventricular remodeling and dysfunction in pressure overload-induced heart failure. Hypertension (2014) 63:1235–40. 10.1161/HYPERTENSIONAHA.114.03279 24688123

[48] Wei JQ Shehadeh L Mitrani JM Pessanha M Slepak TI Webster K Quantitative control of adaptive cardiac hypertrophy by acetyltransferase p300. Circulation (2008) 118:934–46. 10.1161/CIRCULATIONAHA.107.760488 18697823 PMC2726266

[49] Yanazume T Hasegawa K Morimoto T Kawamura T Wada H Matsumori A Cardiac p300 is involved in myocyte growth with decompensated heart failure. Mol Cell Biol (2003) 23:3593–606. 10.1128/MCB.23.10.3593-3606.2003 12724418 PMC154243

[50] Zhang S Weinheimer C Courtois M Kovacs A Zhang CE Cheng AM The role of the Grb2-p38 MAPK signaling pathway in cardiac hypertrophy and fibrosis. J Clin Invest (2003) 111:833–41. 10.1172/JCI16290 12639989 PMC153766

[51] Krishnan J Suter M Windak R Krebs T Felley A Montessuit C Activation of a HIF1alpha-PPARgamma axis underlies the integration of glycolytic and lipid anabolic pathways in pathologic cardiac hypertrophy. Cell Metab (2009) 9:512–24. 10.1016/j.cmet.2009.05.005 19490906

[52] Lairez O Cognet T Schaak S Calise D Guilbeau-Frugier C Parini A Role of serotonin 5-HT2A receptors in the development of cardiac hypertrophy in response to aortic constriction in mice. J Neural Transm (2013) 120:927–35. 10.1007/s00702-013-1011-3 23543114

[53] Jiang D-S Liu Y Zhou H Zhang Y Zhang X-D Zhang X-F Interferon regulatory factor 7 functions as a novel negative regulator of pathological cardiac hypertrophy. Hypertension (2014) 63:713–22. 10.1161/HYPERTENSIONAHA.113.02653 24396025 PMC5349187

[54] Purcell NH Tang G Yu C Mercurio F DiDonato JA Lin A. Activation of NF-kappa B is required for hypertrophic growth of primary rat neonatal ventricular cardiomyocytes. Proc Natl Acad Sci U S A (2001) 98:6668–73. 10.1073/pnas.111155798 11381115 PMC34410

[55] Clemente CFMZ Tornatore TF Theizen TH Deckmann AC Pereira TC Lopes-Cendes I Targeting focal adhesion kinase with small interfering RNA prevents and reverses load-induced cardiac hypertrophy in mice. Circ Res (2007) 101:1339–48. 10.1161/CIRCRESAHA.107.160978 17947798

[56] Métrich M Lucas A Gastineau M Samuel J-L Heymes C Morel E Epac mediates beta-adrenergic receptor-induced cardiomyocyte hypertrophy. Circ Res (2008) 102:959–65. 10.1161/CIRCRESAHA.107.164947 18323524

[57] Wu X Cao Y Nie J Liu H Lu S Hu X Genetic and pharmacological inhibition of Rheb1-mTORC1 signaling exerts cardioprotection against adverse cardiac remodeling in mice. Am J Pathol (2013) 182:2005–14. 10.1016/j.ajpath.2013.02.012 23567640

[58] Manso AM Li R Monkley SJ Cruz NM Ong S Lao DH Talin1 has unique expression versus talin 2 in the heart and modifies the hypertrophic response to pressure overload. J Biol Chem (2013) 288:4252–64. 10.1074/jbc.M112.427484 23266827 PMC3567677

[59] DeBosch B Treskov I Lupu TS Weinheimer C Kovacs A Courtois M Akt1 is required for physiological cardiac growth. Circulation (2006) 113:2097–104. 10.1161/CIRCULATIONAHA.105.595231 16636172

[60] Matsui T Li L Wu JC Cook SA Nagoshi T Picard MH Phenotypic spectrum caused by transgenic overexpression of activated Akt in the heart. J Biol Chem (2002) 277:22896–901. 10.1074/jbc.M200347200 11943770

[61] Chen L Hahn H Wu G Chen CH Liron T Schechtman D Opposing cardioprotective actions and parallel hypertrophic effects of delta PKC and epsilon PKC. Proc Natl Acad Sci U S A (2001) 98:11114–9. 10.1073/pnas.191369098 11553773 PMC58692

[62] Baurand A Zelarayan L Betney R Gehrke C Dunger S Noack C Beta-catenin downregulation is required for adaptive cardiac remodeling. Circ Res (2007) 100:1353–62. 10.1161/01.RES.0000266605.63681.5a 17413044

[63] Yang K-C Jay PY McMullen JR Nerbonne JM. Enhanced cardiac PI3Kα signalling mitigates arrhythmogenic electrical remodelling in pathological hypertrophy and heart failure. Cardiovasc Res (2012) 93:252–62. 10.1093/cvr/cvr283 22038742 PMC3258651

[64] McMullen JR Shioi T Zhang L Tarnavski O Sherwood MC Kang PM Phosphoinositide 3-kinase(p110alpha) plays a critical role for the induction of physiological, but not pathological, cardiac hypertrophy. Proc Natl Acad Sci U S A (2003) 100:12355–60. 10.1073/pnas.1934654100 14507992 PMC218762

[65] Haq S Kilter H Michael A Tao J O’Leary E Sun XM Deletion of cytosolic phospholipase A2 promotes striated muscle growth. Nat Med (2003) 9:944–51. 10.1038/nm891 12808451

[66] Ojamaa K. Signaling mechanisms in thyroid hormone-induced cardiac hypertrophy. Vascul Pharmacol (2010) 52:113–9. 10.1016/j.vph.2009.11.008 20005976 PMC2830872

[67] Kinugawa K Yonekura K Ribeiro RC Eto Y Aoyagi T Baxter JD Regulation of thyroid hormone receptor isoforms in physiological and pathological cardiac hypertrophy. Circ Res (2001) 89:591–8. 10.1161/hh1901.096706 11577024

[68] Lee HW Klein LE Raser J Eghbali-Webb M. An activator protein-1 (AP-1) response element on pro alpha1(l) collagen gene is necessary for thyroid hormone-induced inhibition of promoter activity in cardiac fibroblasts. J Mol Cell Cardiol (1998) 30:2495–506. 10.1006/jmcc.1998.0811 9925384

[69] Ghose Roy S Mishra S Ghosh G Bandyopadhyay A. Thyroid hormone induces myocardial matrix degradation by activating matrix metalloproteinase-1. Matrix Biol (2007) 26:269–79. 10.1016/j.matbio.2006.12.005 17275272

[70] Grueter C van Rooij E Johnson B DeLeon S. A cardiac microRNA governs systemic energy homeostasis by regulation of MED13. Cell (2012) 149:671–83. 10.1016/j.cell.2012.03.029 22541436 PMC3340581

[71] Ito M Roeder RG. The TRAP/SMCC/mediator complex and thyroid hormone receptor function. Trends Endocrinol Metab (2001) 12:127–34. 10.1016/S1043-2760(00)00355-6 11306338

[72] Malik S Roeder RG. Dynamic regulation of pol II transcription by the mammalian mediator complex. Trends Biochem Sci (2005) 30:256–63. 10.1016/j.tibs.2005.03.009 15896744

[73] Martinelli NC Cohen CR Santos KG Castro M Biolo A Frick L An analysis of the global expression of microRNAs in an experimental model of physiological left ventricular hypertrophy. PLoS One (2014) 9:e93271. 10.1371/journal.pone.0093271 24751578 PMC3994002

[74] Kress E Samarut J Plateroti M. Thyroid hormones and the control of cell proliferation or cell differentiation: paradox or duality? Mol Cell Endocrinol (2009) 313:36–49. 10.1016/j.mce.2009.08.028 19737599

[75] López M Alvarez CV Nogueiras R Diéguez C. Energy balance regulation by thyroid hormones at central level. Trends Mol Med (2013) 19:418–27. 10.1016/j.molmed.2013.04.004 23707189

[76] Anderson ME. CaMKII and a failing strategy for growth in heart. J Clin Invest (2009) 119:1082–5. 10.1172/JCI39262 19422097 PMC2673844

[77] Sano M Minamino T Toko H Miyauchi H Orimo M Qin Y p53-induced inhibition of Hif-1 causes cardiac dysfunction during pressure overload. Nature (2007) 446:444–8. 10.1038/nature05602 17334357

[78] Zhang T Johnson EN Gu Y Morissette MR Sah VP Gigena MS The cardiac-specific nuclear delta(B) isoform of Ca2+/calmodulin-dependent protein kinase II induces hypertrophy and dilated cardiomyopathy associated with increased protein phosphatase 2A activity. J Biol Chem (2002) 277:1261–7. 10.1074/jbc.M108525200 11694533

[79] Zhang R Khoo MSC Wu Y Yang Y Grueter CE Ni G Calmodulin kinase II inhibition protects against structural heart disease. Nat Med (2005) 11:409–17. 10.1038/nm1215 15793582

[80] Zhang W Qi F Chen D-Q Xiao W-Y Wang J Zhu W-Z. Ca2+/calmodulin-dependent protein kinase IIdelta orchestrates G-protein-coupled receptor and electric field stimulation-induced cardiomyocyte hypertrophy. Clin Exp Pharmacol Physiol (2010) 37:795–802. 10.1111/j.1440-1681.2010.05382.x 20374261

[81] Hayata N Fujio Y Yamamoto Y Iwakura T Obana M Takai M Connective tissue growth factor induces cardiac hypertrophy through Akt signaling. Biochem Biophys Res Commun (2008) 370:274–8. 10.1016/j.bbrc.2008.03.100 18375200

[82] Schaub MC Hefti M Harder B Eppenberger HM. Various hypertrophic stimuli induce distinct phenotypes in cardiomyocytes. J Mol Med (Berl) (1997) 75:901–20. 10.1007/s001090050182 9428623

[83] Shiojima I Sato K Izumiya Y Schiekofer S Ito M Liao R Disruption of coordinated cardiac hypertrophy and angiogenesis contributes to the transition to heart failure. J Clin Invest (2005) 115:2108–18. 10.1172/JCI24682 16075055 PMC1180541

[84] Shiojima I Walsh K. Regulation of cardiac growth and coronary angiogenesis by the Akt/PKB signaling pathway. Genes Dev (2006) 20:3347–65. 10.1101/gad.1492806 17182864

[85] Chaanine AH Hajjar RJ. AKT signalling in the failing heart. Eur J Heart Fail (2011) 13:825–9. 10.1093/eurjhf/hfr080 21724622 PMC3143831

[86] Warner MH Beckett GJ. Mechanisms behind the non-thyroidal illness syndrome: an update. J Endocrinol (2010) 205:1–13. 10.1677/JOE-09-0412 20016054

[87] Pantos C Dritsas A Mourouzis I Dimopoulos A Karatasakis G Athanassopoulos G Thyroid hormone is a critical determinant of myocardial performance in patients with heart failure: potential therapeutic implications. Eur J Endocrinol (2007) 157:515–20. 10.1530/EJE-07-0318 17893267

[88] Pingitore A Landi P Taddei MC Ripoli A L’Abbate A Iervasi G. Triiodothyronine levels for risk stratification of patients with chronic heart failure. Am J Med (2005) 118:132–6. 10.1016/j.amjmed.2004.07.052 15694896

[89] Olivares EL Marassi MP Fortunato RS da Silva ACM Costa-e-Sousa RH Araújo IG Thyroid function disturbance and type 3 iodothyronine deiodinase induction after myocardial infarction in rats a time course study. Endocrinology (2007) 148:4786–92. 10.1210/en.2007-0043 17628010

[90] Wassen FWJS Schiel AE Kuiper GGJM Kaptein E Bakker O Visser TJ Induction of thyroid hormone-degrading deiodinase in cardiac hypertrophy and failure. Endocrinology (2002) 143:2812–5. 10.1210/endo.143.7.8985 12072417

[91] Van Rooij E Marshall WS Olson EN. Toward microRNA-based therapeutics for heart disease: the sense in antisense. Circ Res (2008) 103:919–28. 10.1161/CIRCRESAHA.108.183426 18948630 PMC2725407

[92] Latronico MVG Condorelli G. Therapeutic use of microRNAs in myo- cardial diseases. Curr Heart Fail Rep (2011) 8:193–7. 10.1007/s11897-011-0068-2 21713604

