# Supplementary material for: Thyroid Hormone-Regulated Cardiac microRNAs are Predicted to Suppress Pathological Hypertrophic Signaling
Source: Front Endocrinol (Lausanne). 2014 Oct 20;5:171. doi: 10.3389/fendo.2014.00171 (PMC4202793; doi:10.3389/fendo.2014.00171)
Supplement: Supplementary file 1 [file Table1.PDF]

Supplemental Table S1

Assay ID	RQ	p-value	Assay ID	RQ	p-value	Assay ID	RQ	p-value
mmu-miR-1905	0.06	0.004	mmu-miR-148a	0.63	0.001	mmu-miR-152	0.82	0.013
mmu-miR-423-5p	0.19	0.020	mmu-miR-127	0.63	0.001	hsa-miR-340	0.84	0.032
mmu-miR-721	0.21	0.006	rno-miR-664	0.64	0.001	mmu-miR-337-5p	0.84	0.012
mmu-miR-463*	0.22	0.001	mmu-miR-328	0.64	0.025	hsa-miR-421	0.88	0.044
mmu-miR-465b-5p	0.24	0.022	mmu-miR-208b	0.64	0.003	mmu-miR-101b	1.33	0.039
mmu-miR-1939	0.26	0.021	mmu-miR-125b-5p	0.64	0.001	hsa-miR-671-5p	1.47	0.029
mmu-miR-590-5p	0.26	0.007	mmu-miR-451	0.65	0.009	hsa-miR-223	1.48	0.000
mmu-miR-2183	0.30	0.000	mmu-miR-497	0.66	0.013	mmu-miR-547	1.49	0.035
mmu-miR-302a	0.30	0.036	mmu-miR-107	0.66	0.019	mmu-miR-93	1.51	0.001
mmu-miR-465c-5p	0.33	0.036	mmu-miR-136	0.66	0.032	mmu-miR-20a*	1.54	0.031
mmu-miR-1944	0.34	0.008	mmu-miR-218	0.66	0.016	mmu-miR-186	1.55	0.043
mmu-miR-322	0.36	0.014	mmu-miR-135b	0.67	0.004	mmu-miR-805	1.66	0.015
mmu-miR-181c	0.38	0.031	mmu-miR-322	0.67	0.012	mmu-miR-532-5p	1.67	0.014
mmu-miR-455	0.41	0.002	mmu-miR-106b	0.67	0.044	mmu-miR-223	1.69	0.000
mmu-miR-673	0.42	0.028	mmu-miR-199b	0.67	0.008	mmu-miR-877*	1.77	>0.0001
mmu-miR-1897-5p	0.42	0.009	mmu-miR-324-3p	0.67	0.000	mmu-miR-132	1.78	0.006
rno-miR-148b-5p	0.45	0.023	mmu-miR-126-5p	0.67	0.000	mmu-miR-1191	1.80	0.013
mmu-miR-504	0.45	0.042	mmu-miR-145	0.68	0.000	rno-miR-146b	1.84	0.001
mmu-miR-190	0.45	0.000	hsa-miR-214	0.68	0.000	mmu-miR-712	1.85	0.009
mmu-miR-509-3p	0.45	0.036	mmu-miR-1954	0.69	0.040	rno-miR-99a*	2.03	0.005
mmu-miR-188-5p	0.47	0.003	mmu-miR-1	0.70	0.039	mmu-miR-34b-3p	2.05	0.016
mmu-miR-221	0.48	0.002	mmu-miR-350	0.70	0.021	mmu-miR-1198	2.06	0.018
mmu-miR-470*	0.48	0.019	mmu-miR-30a	0.70	0.006	mmu-miR-146a	2.21	0.000
mmu-miR-29b	0.48	0.001	mmu-miR-31	0.70	0.003	mmu-miR-484	2.23	0.003
mmu-miR-1928	0.51	0.002	mmu-miR-30c	0.71	0.005	hsa-miR-99b*	2.25	0.013
mmu-miR-324-5p	0.51	0.000	mmu-miR-384-5p	0.71	0.005	mmu-miR-142-5p	2.25	0.010
mmu-miR-193b	0.52	0.000	mmu-miR-195	0.71	0.001	mmu-miR-500	2.37	0.010
mmu-miR-365	0.52	0.007	mmu-miR-1961	0.72	0.038	mmu-miR-18a	2.41	0.004
mmu-miR-135a	0.53	>0.0001	hsa-miR-30e-3p	0.72	0.001	rno-miR-345-3p	2.46	0.003
mmu-miR-26a	0.53	0.000	mmu-miR-720	0.72	0.033	hsa-miR-425	2.50	0.001
mmu-miR-485-3p	0.54	0.044	rno-miR-190b	0.73	0.007	mmu-miR-7b	2.62	0.001
rno-miR-224	0.54	0.001	mmu-miR-30b	0.73	0.004	rno-miR-409-3p	2.88	0.004
mmu-miR-450a-5p	0.54	0.011	mmu-miR-148b	0.73	0.008	mmu-miR-208	3.01	0.001
rno-miR-547	0.56	0.000	mmu-miR-19b	0.73	0.023	mmu-miR-141	3.18	0.007
mmu-miR-335-5p	0.57	0.002	mmu-miR-143	0.74	0.028	mmu-miR-362-5p	3.22	>0.0001
mmu-miR-376c	0.57	0.009	mmu-miR-19a	0.74	0.031	mmu-miR-212	3.25	0.000
mmu-miR-499	0.57	0.002	hsa-miR-200c	0.75	0.048	mmu-miR-124	3.42	0.025
mmu-miR-34a	0.57	0.004	mmu-miR-25	0.75	0.005	mmu-miR-543	3.53	0.020
mmu-miR-224	0.58	0.000	mmu-miR-27a	0.75	0.033	mmu-miR-379	3.82	0.002
mmu-miR-296-5p	0.59	0.023	mmu-miR-376b*	0.75	0.018	mmu-miR-503	4.06	0.003
mmu-miR-92a	0.59	0.000	mmu-miR-29a	0.75	0.038	mmu-miR-449a	4.48	0.029
mmu-miR-181a	0.59	0.015	mmu-miR-30e	0.76	0.014	mmu-miR-1981	4.51	0.021
mmu-miR-345-5p	0.6	0.006	mmu-miR-20a	0.76	0.012	mmu-miR-187	5.36	0.046
hsa-miR-206	0.6	0.004	mmu-miR-434-3p	0.77	0.009	rno-miR-339-3p	7.16	0.036
mmu-miR-322*	0.6	0.002	mmu-miR-340-3p	0.77	0.013	mmu-miR-539	7.97	0.026
hsa-miR-149	0.61	>0.0001	mmu-miR-140	0.77	0.023	mmu-miR-409-3p	8.02	0.000
mmu-let-7c	0.61	0.006	mmu-miR-301a	0.77	0.017	mmu-miR-34c	8.17	0.001
rno-miR-1	0.61	0.010	hsa-miR-30a-3p	0.78	0.002	mmu-miR-28*	10.60	0.000
mmu-miR-450b-3p	0.62	0.008	mmu-miR-503*	0.78	0.032	hsa-miR-27b*	14.15	0.008
mmu-miR-26b	0.62	0.002	hsa-miR-423-3p	0.81	0.014	mmu-miR-674	20.95	0.003
mmu-miR-31*	0.62	0.000	mmu-miR-331-3p	0.81	0.037	mmu-miR-511	62.07	>0.0001
mmu-miR-150	0.62	0.001	mmu-miR-186*	0.81	0.013			

Differentially expressed miRNAs in hyperthyroid LV.
The expression data of 641 known mouse miRNA were obtained with Taqman Megaplex arrays (v3. A and B, rodent) and analyzed using RQ-manager (v1.2) and DataAssist (v3.0). MmamU6 and snoRNA-202 were the most stable miRNA and therefore chosen as endogenous controls. In total 149 miRNAs were differentially expressed ($p < 0.05$). This list included 45 upregulated and 104 downregulated miRNAs. RQ, Relative Quantification.
