# Supplementary material for: Thyroid Hormone-Regulated Cardiac microRNAs are Predicted to Suppress Pathological Hypertrophic Signaling
Source: Front Endocrinol (Lausanne). 2014 Oct 20;5:171. doi: 10.3389/fendo.2014.00171 (PMC4202793; doi:10.3389/fendo.2014.00171)
Supplement: Supplementary file 2 [file Table2.PDF]

Supplemental Table S2

miRNA ID	seed sequence
Upregulated	
miR-124-3p	AAGGCAC
miR-132/212-3p	AACAGUC
miR-141-3p	AACACUG
miR-142-5p	AUAAAGU
miR-146a-5p	GAGAACU
miR-187-3p	CGUGUCU
miR-18a-5p	AAGGUGC
miR-208a-3p	UAAGACG
miR-28-3p	ACUAGAU
miR-339-3p	GAGCGCC
miR-34a/b/c-5p	GGCAGUG
miR-34a/b/c-3p	AUCACUA
miR-362-5p	AUCCUUG
miR-379-5p	GGUAGAC
miR-409-3p	AAUGUUG
miR-484	CAGGCUC
miR-503-5p	AGCAGCG
miR-539-5p	GAGAAAU
miR-543-3p	AACAUUC
miR-7a/b-5p	GGAAGAC
Downregulated	
miR-130a-3p/721	AGUGCAA
miR-181a/c-5p	ACAUUCA
miR-188-5p	AUCCCUU
miR-190a-5p	GAUAUGU
miR-21/590-5p	AGCUUAU
miR-221-3p	GCUACAU
miR-291/302a-3p	AAGUGCU
miR-29b-3p	AGCACCA
miR-423-5p	GAGGGGC
miR-455-3p	CAGUCCA
miR-504-5p	GACCCUG

IPA targeting information.
According to IPA, 31 of the 52 differentially expressed miRNAs had targeting information resulting in 3274 confirmed or highly predicted target mRNAs. Prediction was based on 7-mer seed regions as is presented in this table.
